# Combination of eribulin plus AKT inhibitor evokes synergistic cytotoxicity in soft tissue sarcoma cells

**DOI:** 10.1038/s41598-019-42300-z

**Published:** 2019-04-08

**Authors:** Naotaka Hayasaka, Kohichi Takada, Hajime Nakamura, Yohei Arihara, Yutaka Kawano, Takahiro Osuga, Kazuyuki Murase, Shohei Kikuchi, Satoshi Iyama, Makoto Emori, Shintaro Sugita, Tadashi Hasegawa, Akira Takasawa, Koji Miyanishi, Masayoshi Kobune, Junji Kato

**Affiliations:** 1 Department of Medical Oncology, Sapporo Medical University School of Medicine, Sapporo, Japan; 2 Department of Hematology, Sapporo Medical University School of Medicine, Sapporo, Japan; 3 Department of Orthopedic Surgery, Sapporo Medical University School of Medicine, Sapporo, Japan; 4 Department of Surgical Pathology, Sapporo Medical University School of Medicine, Sapporo, Japan; 5 Department of Molecular and Cellular Pathology, Sapporo Medical University School of Medicine, Sapporo, Japan

## Abstract

An activated AKT pathway underlies the pathogenesis of soft tissue sarcoma (STS), with over-expressed phosphorylated AKT (p-AKT) correlating with a poor prognosis in a subset of STS cases. Recently, eribulin, a microtubule dynamics inhibitor, has demonstrated efficacy and is approved in patients with advanced/metastatic liposarcoma and breast cancer. However, mechanisms of eribulin resistance and/or insensitivity remain largely unknown. In this study, we demonstrated that an increased p-AKT level was associated with eribulin resistance in STS cells. We found a combination of eribulin with the AKT inhibitor, MK-2206, synergistically inhibited STS cell growth *in vivo* as well as *in vitro*. Mechanistically, eribulin plus MK-2206 induced G1 or G2/M arrest by down-regulating cyclin-dependent kinases, cyclins and cdc2, followed by caspase-dependent apoptosis in STS cells. Our findings demonstrate the significance of p-AKT signaling for eribulin-resistance in STS cells and provide a rationale for the development of an AKT inhibitor in combination with eribulin to treat patients with STS.

## Introduction

Soft tissue sarcoma (STS) originating from mesenchymal cells is a rare neoplasm that accounts for around 1% of all adult malignancies^1^. STS comprise a heterogenous group of over 50 recognized histological subtypes^2^. The prognosis for unresectable or metastatic (UM)-STS cases is poor, with an overall survival (OS) of less than 1.2 years after diagnosis^3^. Recommended therapy for most patients with UM-STS is doxorubicin (Dox), a first-line palliative chemotherapy used for over 40 years^4^. Recently, three new drugs, pazopanib^5^, trabectedin^6^ and eribulin^7^, have been introduced for the treatment of UM-STS after Dox therapy. In 2016, eribulin was approved for patients with UM-STS who were previously treated with an anthracycline-containing regimen^7^. Eribulin is a novel microtubule dynamics inhibitor that is a modified analog of halichondrin B^8^. Remarkably, of the three novel therapeutics, only eribulin significantly improved OS compared to dacarbazine. However, outcomes with eribulin for UM-STS may not be enough to fully address unmet clinical needs, in particular for relapsed patients. Therefore, despite the efficacy of eribulin in the disease, the prognosis of UM-STS remains difficult, and there is a need to further develop novel strategies for the treatment of this disease.

Generally, *de novo* and acquired resistances to anti-cancer drugs frequently arise that hamper the results of cancer therapy^9^. However, the mechanisms of eribulin resistance have been largely unknown. Exploring the mechanisms of resistance to eribulin may lead to creating new treatment strategies for UM-STS.

The protein serine/threonine kinase, AKT, also termed protein kinase B, is a pivotal regulator of numerous cellular processes including apoptosis, survival, proliferation and metabolism^10^. Overexpressed p-AKT underlies the pathogenesis of a broad range of human malignancies, including STS, such as leiomyosarcoma, fibrosarcoma, liposarcoma and undifferentiated pleomorphic sarcoma^2,11–13^. Of note, the high expression of p-AKT has been identified as a negative surrogate marker for the survival of patients with STS. Therefore, AKT may represent an attractive target for STS therapeutics.

In the current study, we performed phospho-protein arrays with HT1080 and eribulin resistant HT1080 cell lines to investigate the molecular mechanisms of eribulin resistance in STS cells. This assay revealed that p-AKT levels were up-regulated by eribulin treatment. These observations prompted us to assess the cytotoxicity of eribulin plus AKT inhibitor and whether this combination treatment can overcome eribulin resistance in STS cells. A combination of the AKT inhibitor, MK-2206, with eribulin resulted in synergistically induced G1 or G2/M arrest, followed by apoptosis in STS cells. Overall, our findings provide a clinical framework for using MK-2206 with eribulin to treat patients with UM-STS.

## Results

### Over-expressed p-AKT is associated with eribulin resistance

**Figure 1:**
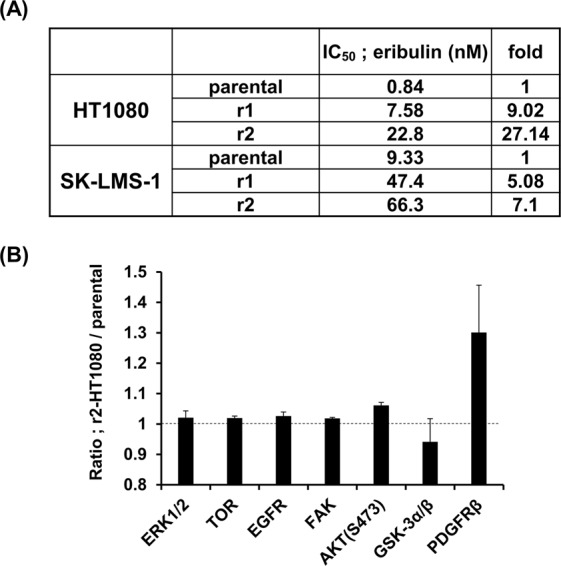
Eribulin resistance in STS cells is associated with up-regulated p-AKT. (**A**) STS cell lines, including eribulin-resistant cell lines (r1 and r2), were cultured in the presence of eribulin for 48 h. Cell proliferation was assessed in triplicate cultures by 3-(4,5-dimethylthiazol-2-yl)-2,5-diphenyl tetrazolium bromide (MTT) assay. The IC_50_ values shown refer to growth inhibition by eribulin. The experiments were repeated three times. (**B**) Immunoblots of protein extracts from parental (HT1080) and r2-HT1080 cell lines were obtained. The phosphorylation status of a panel of proteins was evaluated by image analyzer. The data represents a comparison of parental HT1080 and r2-HT1080 cell lines.

To establish stable STS cell lines with resistance to eribulin, HT1080 (fibrosarcoma) and SK-LMS-1 (leiomyosarcoma) cells were exposed to increasing concentrations of eribulin. Resistant cell lines derived from these cell lines were termed r1/r2-HT1080 and r1/r2-SK-LMS-1, respectively. The resistance of these cells to eribulin compared to parental cells was evaluated with 3-(4,5-dimethylthiazol-2-yl)-2,5-diphenyl tetrazolium bromide (MTT) assays. The IC_50_ values of resistant cells were from 5.08- to 27.14 -fold greater than in the parental cells (Fig. 1A).

**Figure 2:**
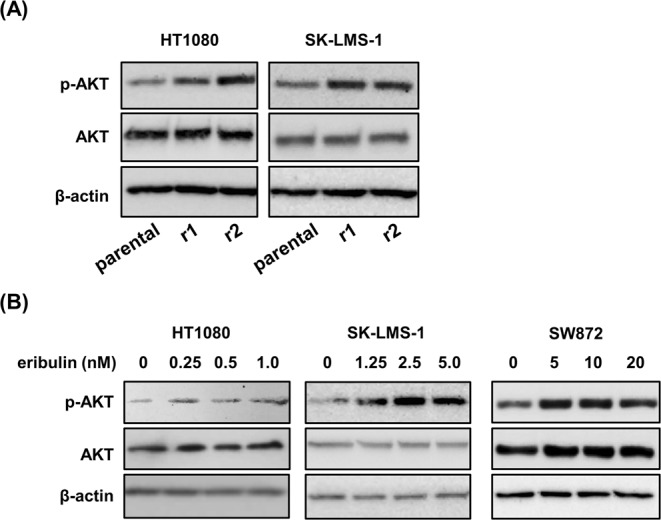
Eribulin treatment promotes phosphorylation of AKT in STS cells. (**A**) Immunoblot of total protein extracts obtained from parental and eribulin-resistant cell lines. The active form of AKT was analyzed using an anti-pAKT (S473) antibody. (**B**) STS cells were treated with increasing dosages of eribulin for 48 h. Whole cell lysates were subjected to immunoblotting using anti–p-AKT, AKT and β-actin antibodies. These experiments were repeated three times.

Growing evidence suggested oncogenes, tumor suppressor genes, and transporter pumps are linked to chemoresistance in numerous cancers^9^. In particular, the activation of oncogenes, such as *PI3K/AKT*^9^, *ERK*^14^, *NF-κB*^15^, *EGFR*^16^ and PDGFR-β^17^, by phosphorylation induced chemoresistance in cancer cells. However, mechanisms of eribulin resistance are less well studied in STS cells. First, we directed our efforts toward screening the phosphorylation status of a panel of kinases, known as oncogenes, using a phospho-kinase array. As shown in Fig. 1B, the expression of PDGFR-β and p-AKT(S473) was increased in r2-HT1080 cells compared to parental cells. This data refers to biological triplicates. We analyzed the result of an array to select druggable targets that can be promptly applied to clinical studies. First, we focused on PDGFR-β, since this is one of the targets of pazopanib, a drug that has already been introduced to patients with STS^5^. We carried out an MTT assay to analyze the cytotoxicity of eribulin plus pazopanib (Supplementary Fig. S1). Unfortunately, this combination did not trigger a synergistic effect in HT1080 cells. The change noted in p-AKT of r2-HT1080 cells was small. We also examined the expression of p-AKT(S473) protein levels in r1/r2-HT1080 and r1/r2-SK-LMS-1 cells by western blotting to confirm the array data. We observed the increased expression of p-AKT(S473) in all eribulin-resistant cell lines (Fig. 2A). Moreover, p-AKT levels were up-regulated, in a dose-dependent fashion, by eribulin treatment for 48 h in parental HT1080, SK-LMS-1 and SW872 (liposarcoma) cell lines (Fig. 2B). These results indicated that eribulin resistance in STS cells may be associated with the phosphorylation of AKT(S473) triggered by eribulin.

### The combination of eribulin and MK-2206 triggers synergistic anti-sarcoma activity

**Figure 3:**
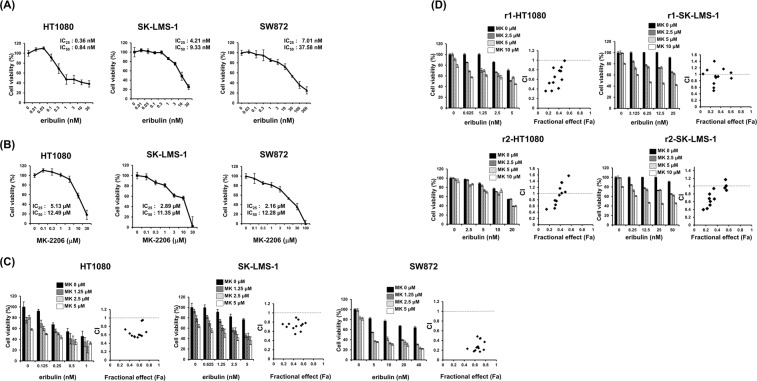
AKT inhibition by MK-2206 synergistically enhances eribulin-induced cytotoxicity in STS cells. (**A**,**B**) HT1080, SK-LMS-1 and SW872 cells were incubated with increasing doses of eribulin and MK-2206 for 48 h. Cell growth reduction was determined using 3-(4,5-dimethylthiazol-2-yl)-2,5-diphenyl tetrazolium bromide (MTT) assays. (**C**,**D**) HT1080, SK-LMS-1 and eribulin resistant cells (r1 and r2) were treated with or without the indicated concentrations of eribulin, MK-2206, or a combination of these for 48 h. The combination of eribulin plus MK-2206 induced synergistic cytotoxicity in even resistant cell lines, although this was less effective than in parental cell lines. The data represents the mean of four independent cultures. Error bars represents the standard deviation (SD). CI: Combination index. CI < 1: synergistic.

Based on our results, we expected that a combination of eribulin plus AKT inhibitor would be effective therapy to overcome eribulin resistance in STS. We chose MK-2206 as an AKT inhibitor in this study since this is an allosteric and highly selective inhibitor^18^, and has been evaluated in several clinical trials^19–22^. Initially, we assessed the cytotoxicity of eribulin and MK-2206, respectively. Eribulin and MK-2206 suppressed the growth of STS cell lines, with values for IC_50_ shown for various cell lines (Fig. 3A,B). Subsequently, we conducted a study of combination treatment using an MTT assay to test whether MK-2206 with eribulin induced increased cytotoxicity compared with each monotherapy. A combination of eribulin plus MK-2206 induced synergistic cytotoxicity in parental STS cell lines at all indicated MK-2206 doses (Fig. 3C), accompanied by the inhibition of AKT phosphorylation (Supplementary Fig. S2). Even in cells resistant to eribulin, a subset of combination doses triggered synergistic cytotoxicity (Fig. 3D). Of note, eribulin plus MK-2206 treatment at IC_50_ values (Fig. 1A and Supplementary Table S1) suppressed cell growth in established STS cell lines with resistance to eribulin (Fig. 3D).

### Eribulin plus MK-2206 promotes G1 or G2/M arrest in STS cells

**Figure 4:**
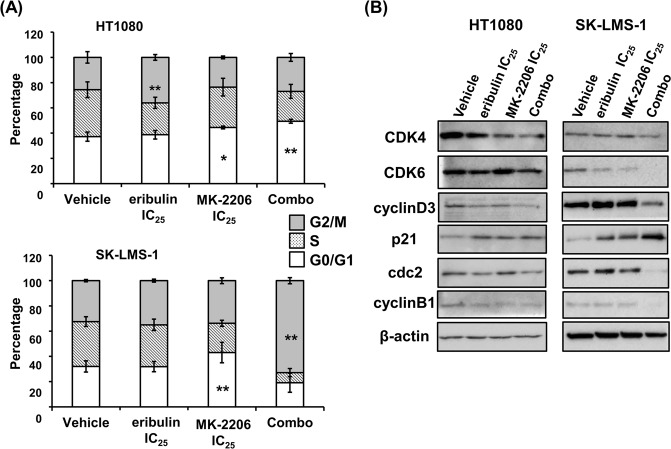
Combined treatment of eribulin plus MK-2206 triggers G1 or G2/M arrest in STS cells. (**A**) HT1080 or SK-LMS-1 cells were untreated (vehicle) or treated with eribulin and/or MK-2206 for 24 h, and then stained with propidium iodide. Subsequently, cell cycles were analyzed by flow cytometry. Error bars represent the standard deviation (SD). *P < 0.05, **P < 0.01. Combo: combined eribulin and MK-2206. (**B**) Immunoblot analysis of cell lysates after treatment of cells with combined eribulin and MK-2206 for 24 h. Cell cycle–regulated proteins were analyzed using the indicated antibodies. The β-actin protein served as a loading control for each experiment. Combo: combined eribulin and MK-2206. These experiments were repeated three times.

To elucidate the cytotoxic mechanisms of eribulin plus MK-2206 in STS cells, we examined the effects of combination treatment on the cell cycle. HT1080 and SK-LMS-1 cell lines were treated with eribulin with or without MK-2206 IC_25_ concentrations, for 24 hours and analyzed by flow cytometry. Eribulin is known to induce G2/M arrest^23^, while MK-2206 promotes G1 arrest in cancer cells^18^. As expected, eribulin significantly induced G2/M arrest in STS cells (Fig. 4A; *P < *0.01), but its effect at the indicated concentrations in SK-LMS-1 cells was of a low power. MK-2206 treatment significantly increased the percentage of cells in the G1 phase and significantly decreased the percentage of cells in the S phase in both cell lines (Fig. 4A; *P < *0.05 for HT1080 and *P < *0.01 for SK-LMS-1). Notably, eribulin plus MK-2206 treatment dramatically induced G1 arrest in HT1080 cells, and G2/M arrest in SK-LMS-1 cells (Fig. 4A; *P < *0.01 for both). To analyze the mechanism of G1 or G2/M arrest induced by eribulin plus MK-2206, we determined protein levels of several cell cycle-related proteins in STS cells using western blotting. The expression of the G1 arrest–associated proteins, CDK4/6 and cyclin D3, were apparently decreased, while p21 expression was increased (Fig. 4B). In terms of G2/M arrest, the expression of cdc2 and cyclin B1 was suppressed simultaneously. These results showed that combination treatment of eribulin plus the AKT inhibitor MK-2206 inhibited the proliferation of STS cells and this was accompanied by G1 or G2/M arrest with the down-regulation of CDK4/6, cdc2 and cyclin B1/D3.

### Eribulin plus MK-2206 treatment induces caspase-dependent apoptosis

**Figure 5:**
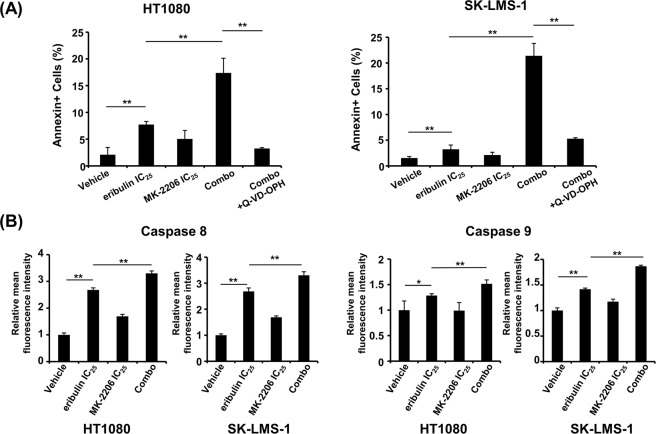
Combination treatment of eribulin plus MK-2206 induces caspase-dependent apoptosis of STS cells. (**A**) HT1080 and SK-LMS-1 cells were treated with IC_25_ doses of eribulin, MK-2206, or a combination of both (combo), with or without a pan-caspase inhibitor, Q-VD-OPH (20 µM), for 48 h. Q-VD-OPH was added 1 h before eribulin and MK-2206 treatments. Apoptotic cells were analyzed by flow cytometry using annexin V/7-AAD staining. Error bars represent the standard deviation (SD). **P < 0.01. (**B**) HT1080 and SK-LMS-1 cells were treated with IC_25_ doses of eribulin, MK-2206, or a combination of both (combo) for 48 h. Activities of caspases 8 and 9 were determined by flow cytometry. These experiments were repeated three times. Error bars represent the SD. *P < 0.05, **P < 0.01.

To investigate whether eribulin plus MK-2206 might also evoke apoptosis in STS cells, we performed flow cytometry analysis. As shown in Fig. 5B, Annexin V/7-AAD staining showed a remarkably higher percentage of apoptosis among HT1080 and SK-LMS-1 cells. Treatment with the pan-caspase inhibitor, Q-VD-OPH, inhibited eribulin plus MK-2206-induced apoptosis. We next assessed whether the apoptosis was mediated via an intrinsic or extrinsic pathway using flow cytometry. The activities of caspases 8 and 9 were significantly up-regulated by eribulin plus MK-2206 treatment compared to each monotherapy cohort (Fig. 5B). These data suggested that eribulin plus MK-2206-induced apoptosis occurred medicated through intrinsic and extrinsic caspase-dependent pathways. Taken together, this combination therapy may be effective for UM-STS patients in a clinical setting.

### Anti-tumor effect of eribulin plus MK-2206 in STS xenograft models

**Figure 6:**
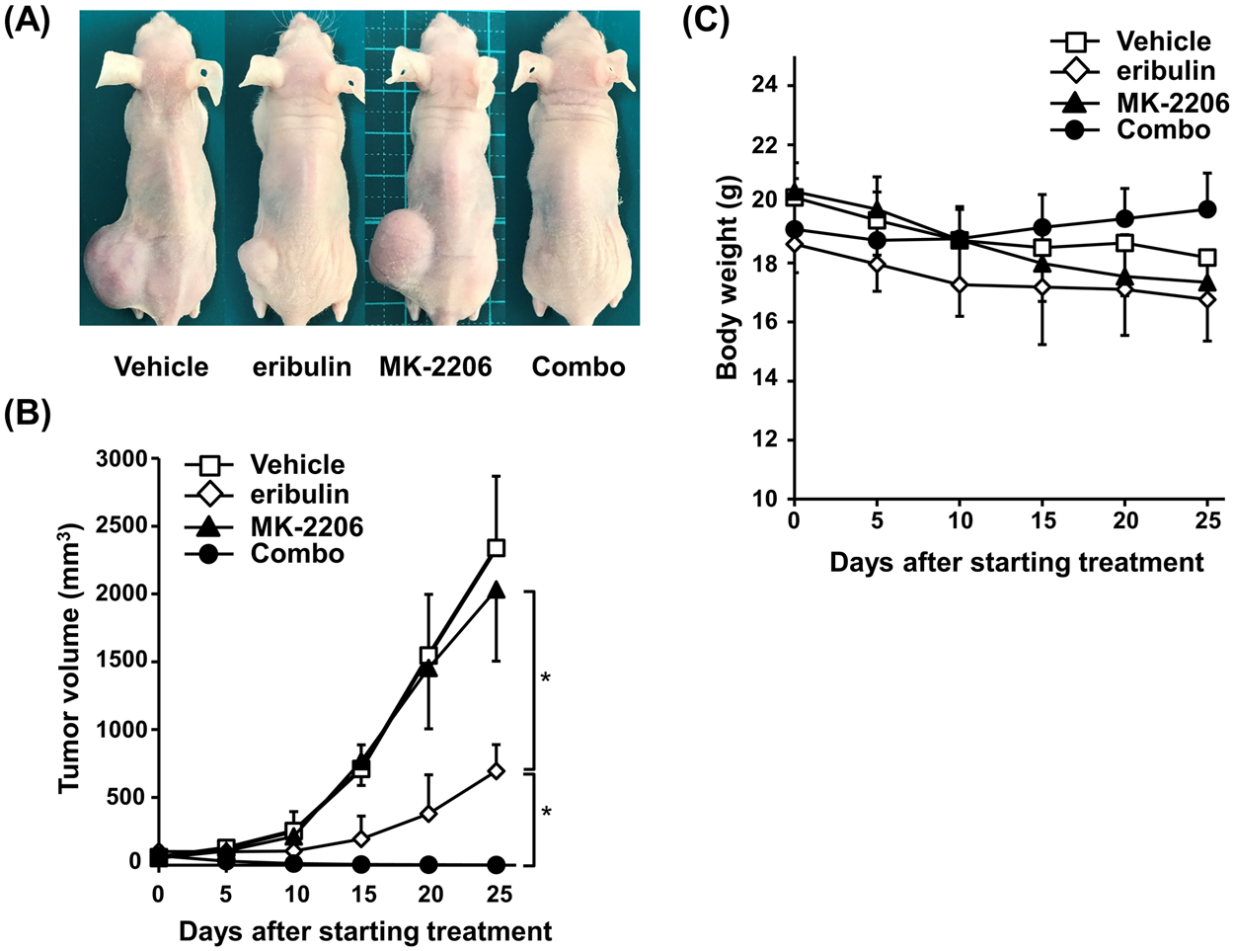
Combination of eribulin and MK-2206 augments tumor suppression in a mouse xenograft STS model. (**A**) Representative images of subcutaneous tumors in each group are shown. (**B**) Tumor volumes were measured and calculated at every treatment schedule. Data are presented as the mean of data ± standard deviation (SD; n = 5). *P < 0.01. (**C**) Mice weights were monitored every 2 days. Data are presented as the mean of data ± SD (n = 5). Combo: combined eribulin and MK-2206.

To explore the clinical potential of eribulin plus MK-2206, we examined the ability of this combination therapy to inhibit STS tumor growth *in vivo* using a subcutaneous HT1080 murine xenograft model. Mice were treated with 0.25 mg/kg eribulin once a week in combination with, or without, 0.12 mg/kg MK-2206 three times per week for three weeks. Tumor burden was significantly suppressed by treatment with eribulin compared with vehicle, whereas mice treated with MK-2206 showed a progressive increase in tumor volume throughout the evaluation period (Fig. 6A,B). When MK-2206 was administered in combination with eribulin, anti-tumor activity was dramatically enhanced as with *in vitro* studies. Strikingly, eribulin plus MK-2206 treatment led to complete responses in 4 of 5 mice, and was well tolerated, without causing significant body weight loss compared to the eribulin alone cohort in this experimental setting (Fig. 6C). No adverse effects in normal tissues were attributable to the administration of eribulin and/or MK-2206 upon necropsy during this study (data not shown). Collectively, these data suggested the possibility of applying this combination for UM-STS treatment.

## Discussion

In the present study, our data demonstrated that AKT plays a key role in the resistance to eribulin by a subset of STS. These observations led us to evaluate combination therapy of eribulin with an AKT inhibitor to combat eribulin resistance and/or insensitivity in STS cells. Eribulin combined with MK-2206 dramatically inhibited STS cell growth *in vivo* as well as *in vitro*, suggesting its clinical application for patients with UM-STS whose prognosis is poor.

Eribulin is approved as a therapeutic for patients with UM-STS who have received a prior anthracycline-containing regimen, with liposarcoma as the indication in the United States and European Union^24^. In Japan, eribulin can be used for all histological types of STS. A phase III trial revealed eribulin treatment significantly prolonged OS for 2 months compared to dacarbazine treatment for L-sarcoma^7^. To further evaluate efficacy in a relapsed/refractory setting, it was reasonable to explore combination therapy of eribulin with other agents. Additionally, identifying mechanisms of eribulin resistance may help in choosing agents to combine with eribulin. We hypothesize that AKT may be an appropriate target that can be applied promptly in clinical trials, with reference to the results of a phospho-kinase array and of previous reports^19–22^. Therefore, we investigated the use of an AKT inhibitor in combination with eribulin to overcome eribulin resistance in STS cells. As expected, this combination therapy synergistically evoked cell-cycle arrest followed by apoptosis *in vitro*, and effectively exerted anti-tumor activity *in vivo* studies of STS cells. These proof-of-concept experiments highlight how eribulin plus an AKT inhibitor is a promising strategy for treating patients with UM-STS. Additionally, determining the expression level of p-AKT may be useful as a biological marker of eribulin responsiveness.

MK-2206 is a highly selective and orally active AKT inhibitor^18^. The efficacy and toxicity of MK-2206 for advanced solid tumors and hematological malignancies is under investigation in a clinical setting. A phase II study revealed that MK-2206 plus erlotinib was effective for advanced non-small cell lung cancer^20^. In addition, two phase II studies for lymphoma and chronic lymphocytic leukemia, respectively, yielded favorable results^19,21^. In terms of STS, one of three leiomyosarcoma patients showed stable disease for four months in a phase I study^22^. The doses of MK-2206 used in the current study were lower than those of previous studies in human cancers, which is encouraging for any future clinical trials.

It has been shown that breast cancer cells that harbor mutated *PTEN* or *PIK3CA* were sensitive to MK-2206^25^. HT1080 and SK-LMS-1 cells that harbor wild-type *PTEN* and *PIK3CA* were resistant to MK-2206 in its clinical therapeutic ranges. Therefore, mutations of *PTEN* and *PIK3CA* may be related to sensitivity to MK-2206 in HT1080 and SK-LMS-1 cell lines. Accumulating evidence demonstrated MK-2206 increased the cytotoxicity of chemotherapeutics among a subset of cancer types, not only in experimental setups^25,26^ but in human studies also^19,20,27^. To our knowledge, this study was the first attempt to find an effective eribulin-based therapy combined with other agents for STS. MK-2206 was found to sensitize cancer cells to DNA-damaging agents^28^. Specifically, MK-2206 cytotoxicity was enhanced when combined with anti-microtubule inhibitors such as eribulin and paclitaxel^29^. As previously reported, our present studies demonstrated that MK-2206 enhanced the eribulin-induced anti-tumor effect. Eribulin combined with MK-2206 induced G1 or G2/M arrest in STS cells. Such different mechanisms of cytotoxicity toward STS cells may depend on the cell type. Notably, this combination therapy evoked an extensive anti-tumor effect *in vivo* compared to *in vitro* studies. One of the plausible mechanisms for the drastic responses observed *in vivo* may be immune responses activated by eribulin treatment^30^. However, further examination is required of eribulin in combination with MK-2206 in clinical studies of UM-STS patients.

In conclusion, this study demonstrated that activated AKT is associated with eribulin resistance in STS cells. Furthermore, eribulin in combination with MK-2206 synergistically suppresses STS growth through G1 or G2/M arrest, and, subsequently, caspase-dependent apoptosis. Our results highlight a potential clinical application for eribulin and MK-2206 combination therapy in the improvement of clinical outcomes in patients with UM-STS.

## Materials and Methods

### Reagents and human cell lines

Eribulin was kindly provided by Eisai Inc. (Tokyo, Japan) and Sapporo Medical University for *in vitro* and *in vivo* use, respectively. MK-2206 was obtained from ChemScene (Monmouth, NJ, USA). Pazopanib was obtained from AdooQ BioScience (Manassas, VA, USA). Stock solutions of these reagents were generated by dissolving the powder in 100% dimethyl sulfoxide (DMSO; Sigma–Aldrich St. Louis, MO, USA) at 10 mM. HT1080, SK-LMS-1 and SW872 cell lines were purchased from ATCC (Manassas, VA, USA). Both cell lines were maintained in DMEM (Sigma–Aldrich) containing 10% fetal bovine serum, 1% penicillin–streptomycin and 2 μM L-glutamine.

### Establishment of eribulin-resistant STS cell lines

Eribulin-resistant STS cell lines were generated by culturing HT1080 and SK-LMS-1 cell lines in stepwise increasing doses (0.1–5.0 nM and 1.0–10 µM, respectively) of eribulin for more than 3 months. Resistant cell lines were named r1/r2-HT1080 and r1/r2-SK-LMS-1, and were continuously cultured in eribulin-containing media.

### Phospho-kinase assay

Alterations in the phosphorylation status of multiple kinases in eribulin resistance compared to parental cells were screened using a Human Phospho-Kinase Array Kit (Proteome Profiler^TM^ Array; R&D Systems, Minneapolis, MD, USA), which contained 43 different kinases. Briefly, cell lysates were prepared and incubated with membranes. Phosphorylated kinases were determined with an anti-phospho antibody. The relative expressions of phosphorylated kinases were quantified with Image J software (Rasband, W.S., ImageJ, U.S. National Institutes of Health, Bethesda, Maryland, USA).

### Cell cycle analysis

STS cells (5 × 10^5^/well) were incubated in medium containing eribulin and/or MK-2206 for 48 h. After collecting floating cells in media, adhesive cells were washed, fixed in ethanol, and stained with propidium iodide using a cell cycle analysis kit (FxCycle PI/RNase Staining Solution; Thermo Fisher Scientific, Waltham, MA, USA) according to the manufacturer’s instructions, followed by analysis on a BD FACS Canto II instrument using FACSDiva. Cell cycle analysis was performed using Flow Jo software (Tree Star Inc.; Ashland, OR, USA).

### Western blot analysis

As previously described^31^, cells were cultured with or without stimuli; cells were then harvested, washed, and lysed. Protein (40 μg) was denatured in SDS buffer and heated for 5 min at 100 °C. Proteins were separated on MULTIGEL II mini gels (COSMO BIO CO., LTD., Tokyo, Japan) and transferred to polyvinylidene fluoride membranes (Immobilon, Merck Millipore Ltd., Burlington, MA, USA). Membranes were blocked using 5% milk in phosphate buffered saline (PBS) for 30 min at RT and incubated overnight at 4 °C with primary antibodies: pAKT (#4058), AKT (#4685), cdk4 (#12790), cdk6(#13331), cyclinD3 (#2936), cyclinE1 (#4129), p21 (#2947), cdc2 (#9116), cyclinB1 (#4138; all from Cell Signaling Technology, Danvers, MA, USA); and actin-HRP (sc-1615; Santa Cruz Biotechnology, Dallas, TX, USA). After washing, stained proteins were visualized using LAS-4000 mini (Fujifilm, Tokyo, Japan).

### Growth inhibition and apoptosis assays

The inhibitory effect of eribulin and MK-2206 on STS cell line growth was assessed by MTT assay. Apoptosis was quantified using an annexin V/7-AAD staining kit (Annexin V PE Apoptosis Detection Kit I; BD Biosciences, San Jose, CA, USA), followed by analysis on a BD FACS Canto II instrument using FACSDiva (BD Biosciences) as previously described^32^. Levels of caspases 8 and 9 were determined by flow cytometric assays using a caspase 8 assay kit (CaspaLux8-L1D2; OncoImmunin, Inc., Gaithersburg, MD, USA) and a caspase 9 assay kit (CaspaLux9-M1D2; OncoImmunin, Inc.), respectively.

### Murine xenograft model of human STS

HT1080 cells were pelleted, resuspended in 100 μL of DMEM with 100 μL of Matrigel (Corning, Corning, NY, USA), and inoculated subcutaneously (5 × 10^5^ cells per mouse) in the left flank into 5-week-old BALB/c-nu mice (n = 5 per cohort). When the tumor diameter reached 5 mm, mice were assigned to four cohorts that were treated with vehicle, MK-2206, or eribulin, with or without MK-2206. Eribulin (0.25 mg/kg) was injected once per week via tail vein, and MK-2206 (0.12 mg/kg), suspended in 30% Captisol was administered by oral gavage three times per week^33^. Tumor volumes were calculated as previously reported^34^. All animal studies were carried out in accordance with the National Institutes of Health guidelines for the use of laboratory animals. All animal protocols were conducted according to the protocol approved by the Animal Ethics Committee of the Sapporo Medical University School of Medicine. After completion of treatment, complete necropsy was performed to evaluate tumor suppressive and adverse effects in xenografts and normal tissues. Excised samples were subjected to hematoxylin and eosin staining.

### Statistical analysis

Statistical significance of differences was determined using Student’s *t* test or ANOVA and subsequent post-hoc tests when the multiple independent variables were compared. Statistical significance was defined as *P* < 0.05. Values for IC_25_ and IC_50_, and the combination index were calculated using CompuSyn software^35^.

## Supplementary information

Supplementary figure, table, information
