# Supplementary material for: Combination of eribulin plus AKT inhibitor evokes synergistic cytotoxicity in soft tissue sarcoma cells
Source: Sci Rep. 2019 Apr 8;9:5759. doi: 10.1038/s41598-019-42300-z (PMC6453888; doi:10.1038/s41598-019-42300-z)

Combination of eribulin plus AKT inhibitor evokes synergistic cytotoxicity in soft tissue sarcoma cells

Naotaka Hayasaka[1*], Kohichi Takada[1,2*¶], Hajime Nakamura[1], Yohei Arihara[1], Yutaka Kawano[1], Takahiro Osuga[1], Kazuyuki Murase[1,2], Shohei Kikuchi[1,2], Satoshi Iyama[2], Makoto Emori[3], Shintaro Sugita[4], Tadashi Hasegawa[4], Akira Takasawa[5], Koji Miyanishi[1], Masayoshi Kobune[2], Junji Kato[1]

[1]Department of Medical Oncology, Sapporo Medical University School of Medicine, Japan

[2]Department of Hematology, Sapporo Medical University School of Medicine, Japan

[3]Department of Orthopedic Surgery, Sapporo Medical University School of Medicine, Japan

[4]Department of Surgical Pathology, Sapporo Medical University School of Medicine, Japan

[5]Department of Molecular and Cellular Pathology, Sapporo Medical University School of Medicine, Japan

[*]These authors contributed equally to this work.

[¶]To whom correspondence should be addressed.

E-mail: ktakada@sapmed.ac.jp

Contact Information

Kohichi Takada, M.D., Ph.D.

Department of Medical Oncology,

Sapporo Medical University School of Medicine

South-1, West-16, Chuo-ku,

Sapporo, Hokkaido, Japan 060-8543

E-mail: ktakada@sapmed.ac.jp

Phone: +81-11-611-2111 (Ext 32540)

Supplementary Figure S1

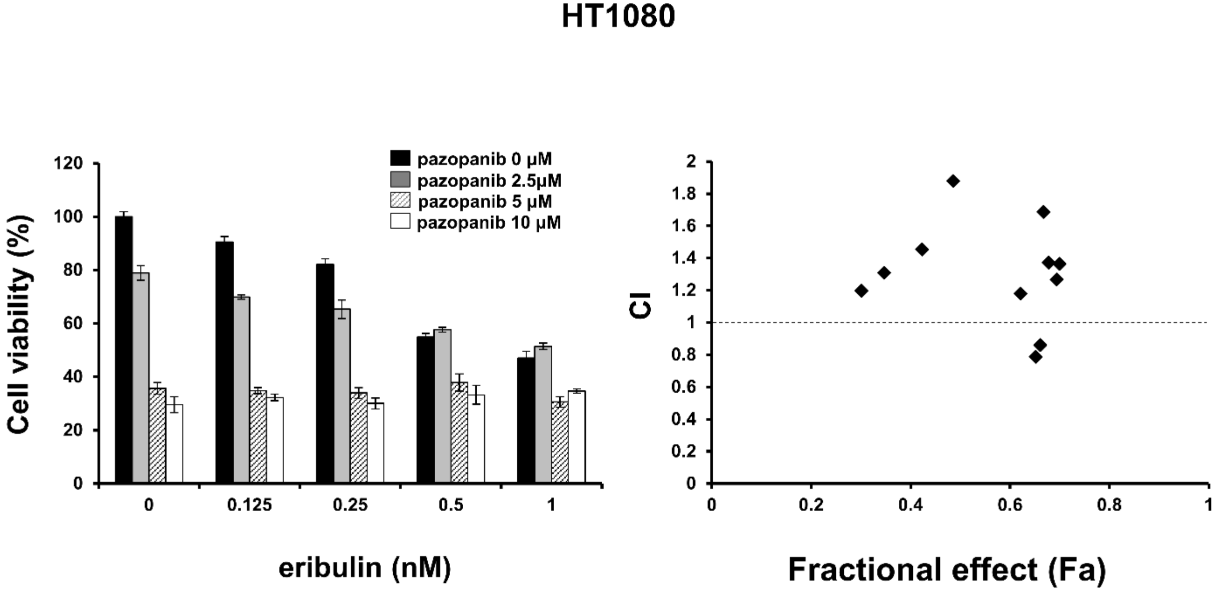

Pazopanib did not synergistically enhance eribulin-induced cytotoxicity in HT1080 cell lines.

HT1080 cell lines was incubated with increasing doses of eribulin and pazopanib for 48 h. Cell growth reduction was determined using 3-(4,5-dimethylthiazol-2-yl)-2,5-diphenyl tetrazolium bromide (MTT) assays. The combination of eribulin plus pazopanib did not induce synergistic cytotoxicity. The data represents the mean of four independent cultures. Error bars represents the standard deviation (SD). CI: Combination index. CI < 1: synergistic.

Supplementary Figure S2

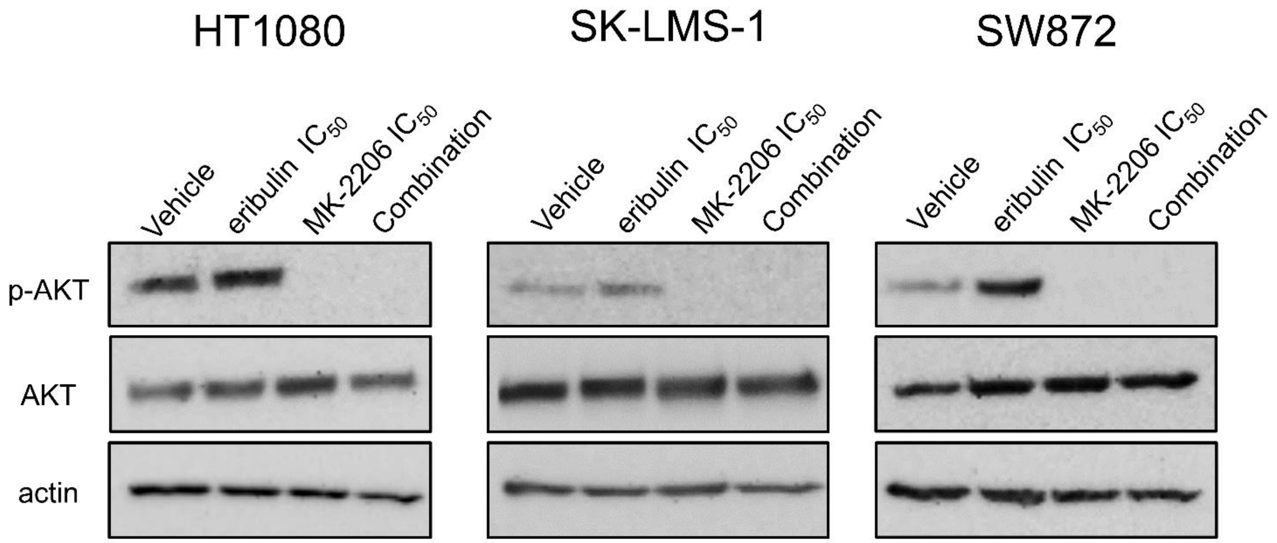

MK-2206 suppressed phosphorylation of AKT in STS cell lines.

We determined protein levels of AKT and phosphorylated AKT in STS cells using western blotting. MK-2206 completely suppressed the expression of p-AKT in both monotherapy and combination therapy.

Supplementary Table S1

		IC_{50} ; MK-2206 (μM)	fold
HT1080	parental	12.49	1
	r1	20.39	1.62
	r2	23.22	1.85
SK-LMS-1	parental	11.35	1
	r1	18.21	1.6
	r2	20.58	1.81

IC_{50} values for parental and eribulin-resistant cell lines

Uncropped western blots used in Figure 2, Figure 4, and Supplementary Figure 2.

Figure 2
(A)

HT1080

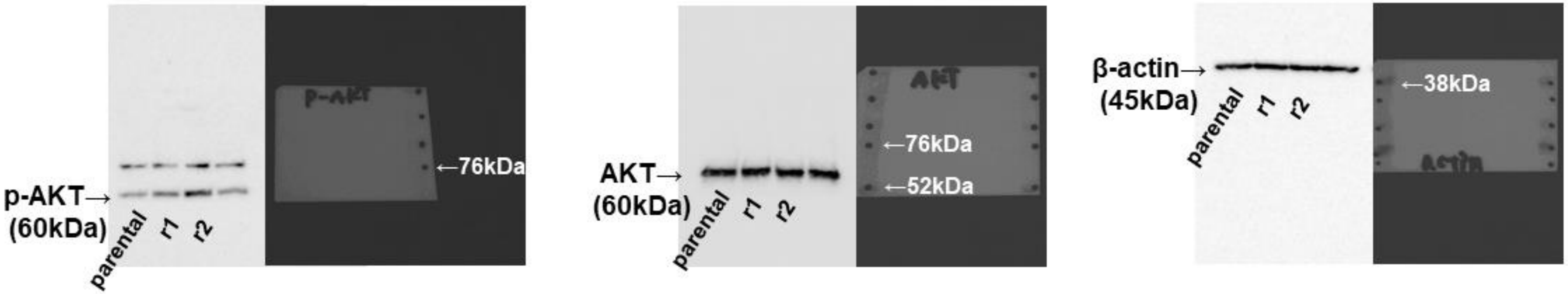

SK-LMS-1

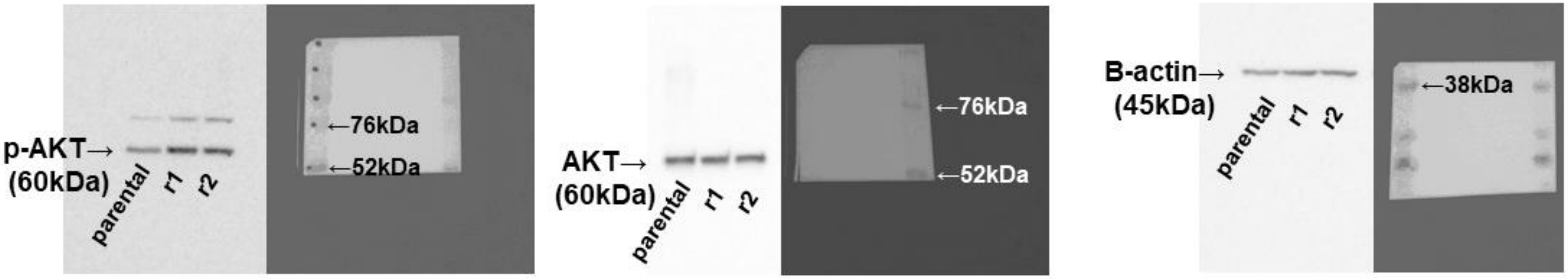

Figure 2
(B)

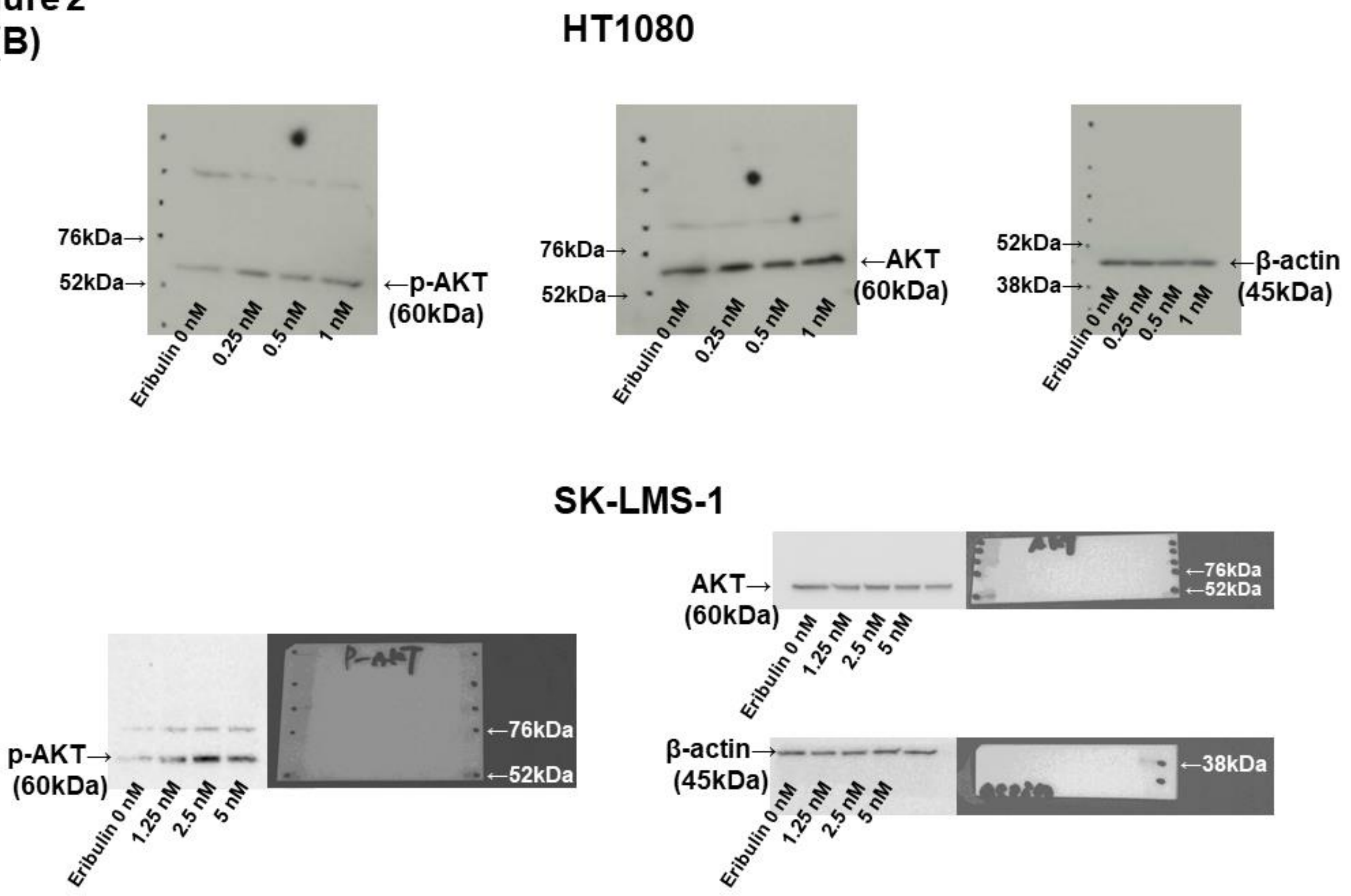
HT1080
76kDa→
52kDa→
←p-AKT
(60kDa)
Eribulin 0 nM
0.25 nM
0.5 nM
1 nM
76kDa→
52kDa→
←AKT
(60kDa)
Eribulin 0 nM
0.25 nM
0.5 nM
1 nM
52kDa→
38kDa→
←β-actin
(45kDa)
Eribulin 0 nM
0.25 nM
0.5 nM
1 nM
SK-LMS-1
p-AKT→
(60kDa)
Eribulin 0 nM
1.25 nM
2.5 nM
5 nM
←76kDa
←52kDa
AKT→
(60kDa)
Eribulin 0 nM
1.25 nM
2.5 nM
5 nM
←76kDa
←52kDa
β-actin→
(45kDa)
Eribulin 0 nM
1.25 nM
2.5 nM
5 nM
←38kDa

Figure 2
(B)

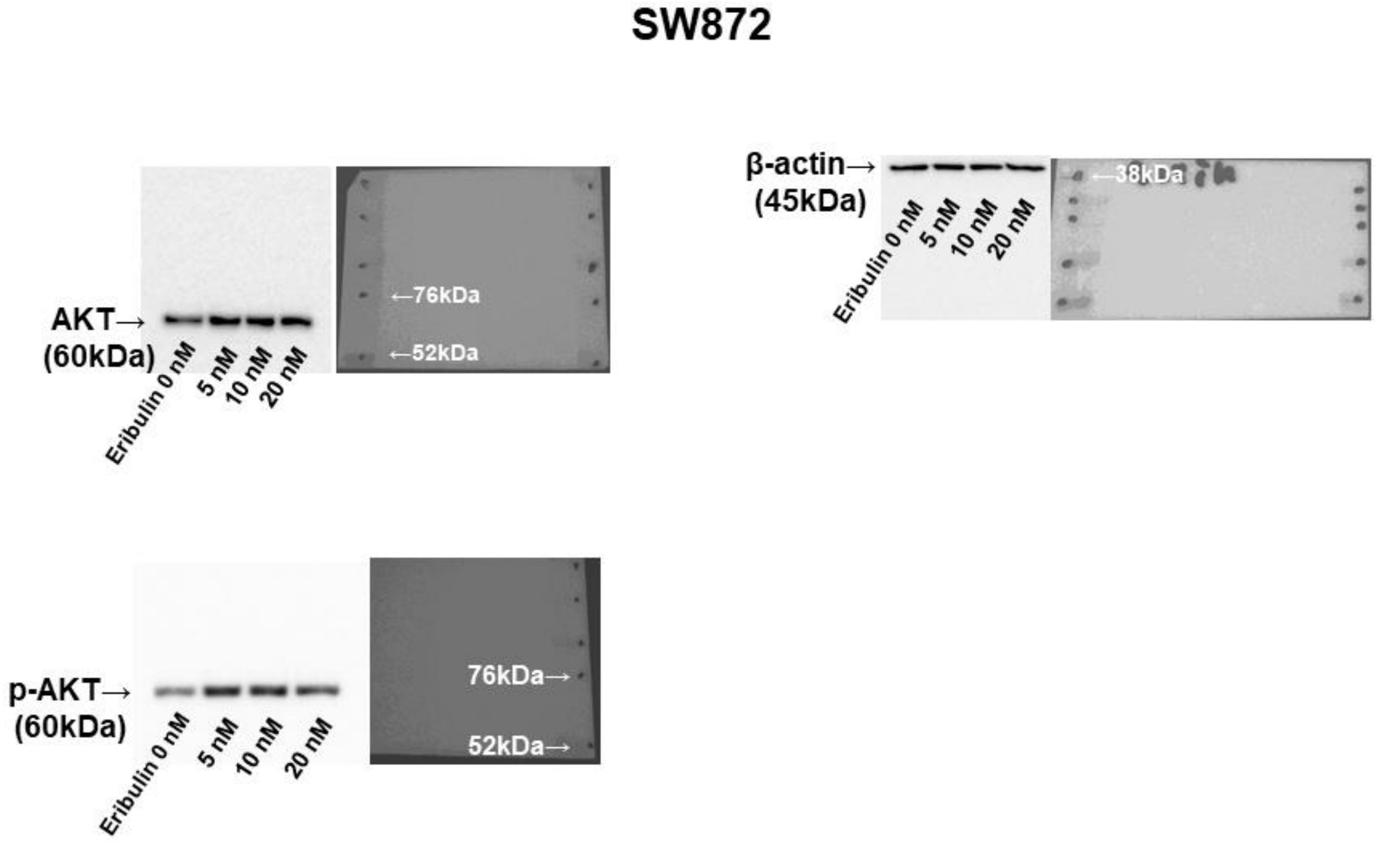
SW872
AKT→
(60kDa)
Eribulin 0 nM
5 nM
10 nM
20 nM
←76kDa
←52kDa
β-actin→
(45kDa)
←38kDa
p-AKT→
(60kDa)
76kDa→
52kDa→

Figure 4 (B)

HT1080

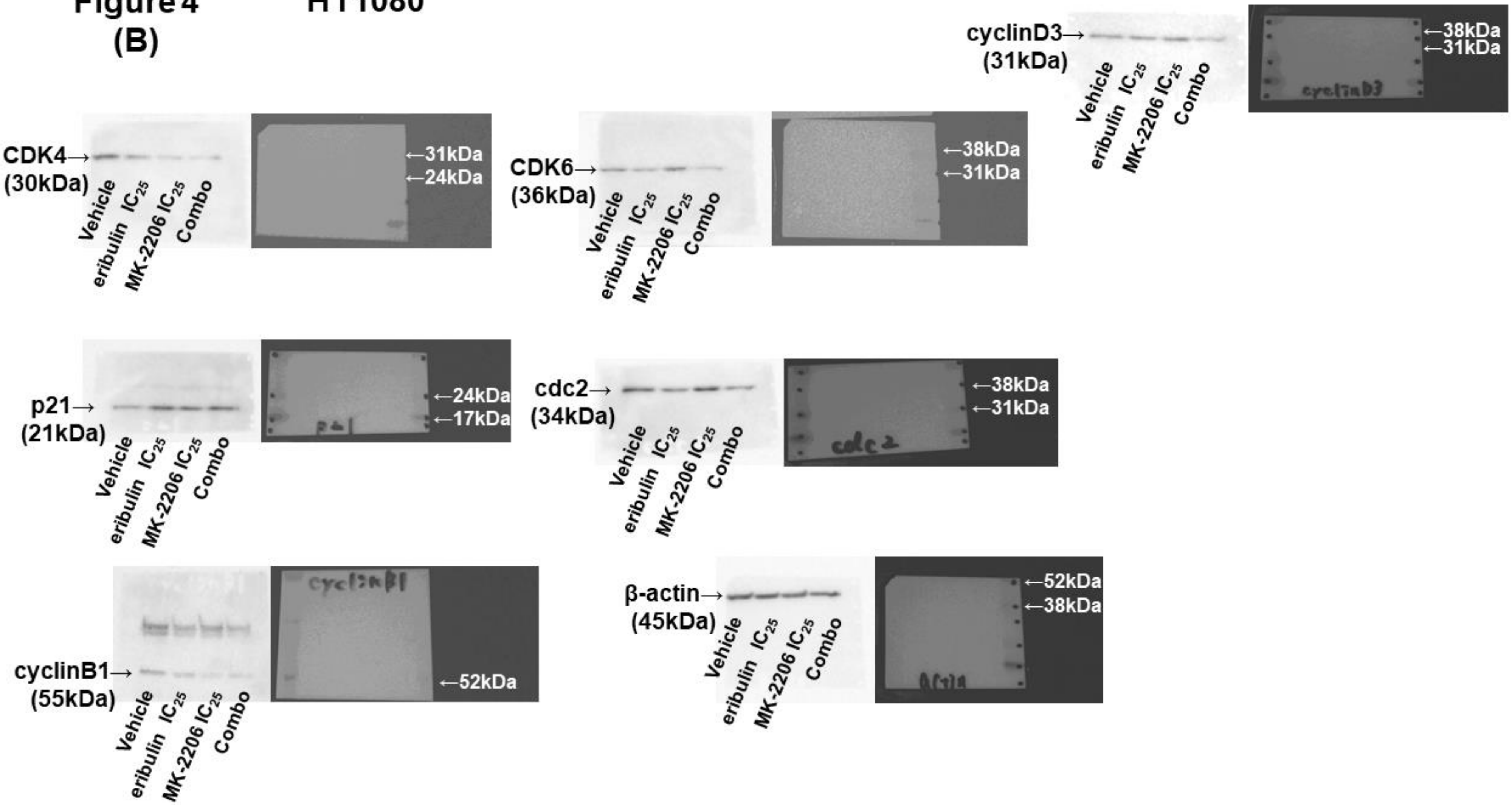

Figure 4 (B) SK-LMS-1

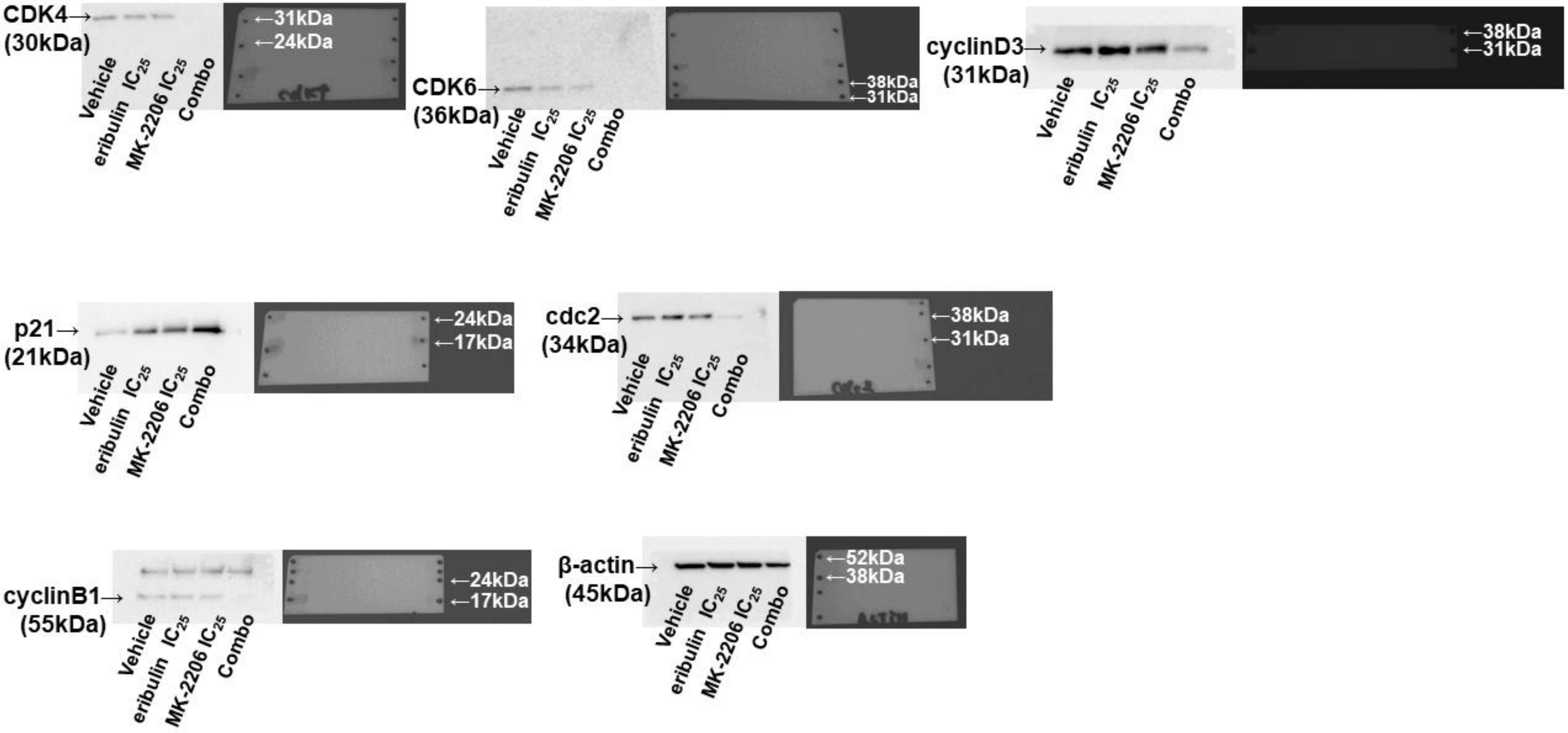

Supplementary Figure S2

HT1080

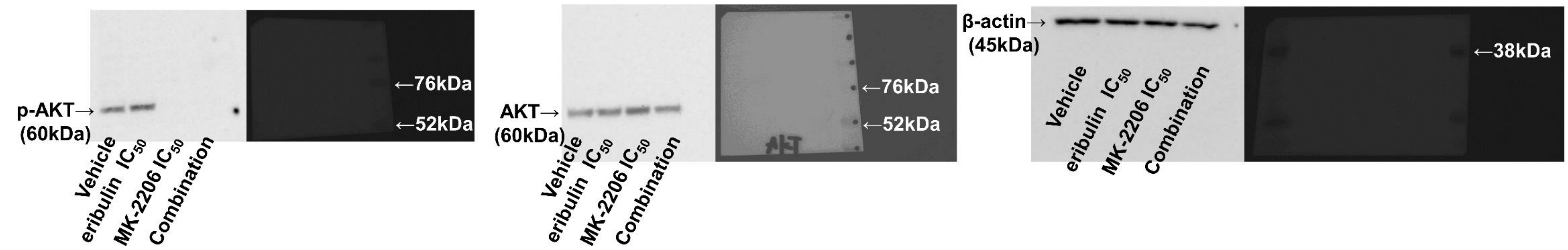

SK-LMS-1

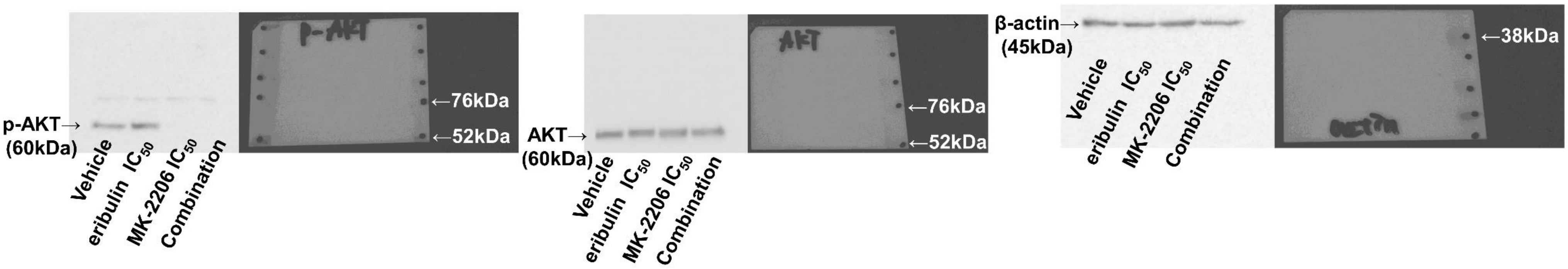

Supplementary Figure S2

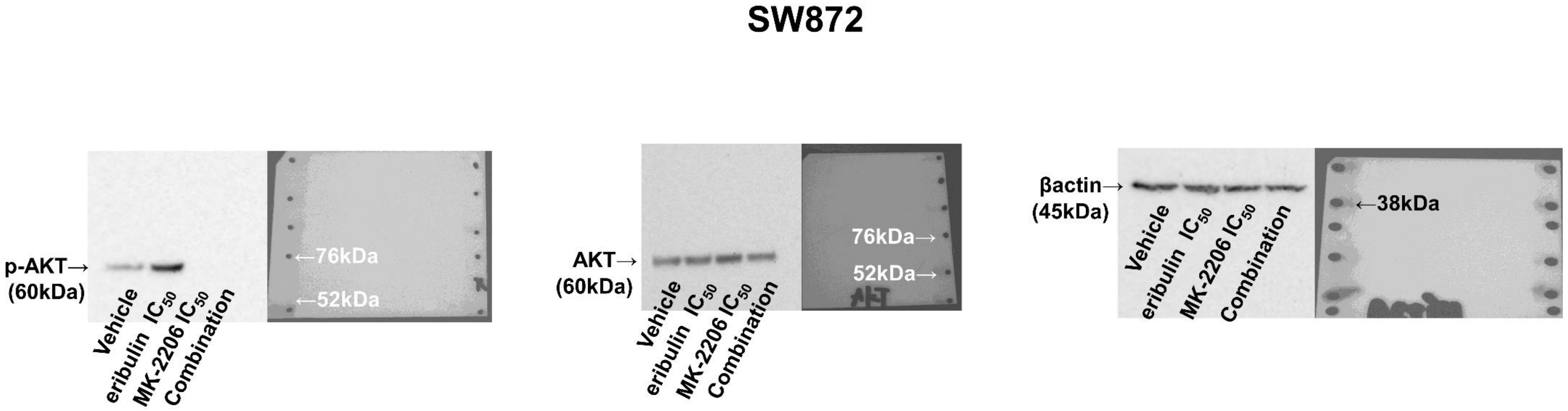

Supplement: Supplementary file 1 — Supplementary figure, table, information [file 41598_2019_42300_MOESM1_ESM.pdf]
